# MRD in multiple myeloma: more questions than answers?

**DOI:** 10.1038/s41408-017-0028-5

**Published:** 2017-12-05

**Authors:** Philippe Moreau, Elena Zamagni

**Affiliations:** 1 University Hospital, Nantes, France; 2 Istituto di Ematologia Seragnoli, Bologna, Italy

The growing interest in minimal residual disease (MRD) assessment in multiple myeloma (MM) is related to the high quality of responses achieved with novel agents and to the development of reliable techniques to evaluate MRD both within the bone marrow using next-generation sequencing (NGS) or next-generation flow cytometry (NGF), and outside the bone marrow using imaging techniques, such as positron emission tomography-computed tomography (PET-CT)^1^. A consensus paper by the International Myeloma Working Group (IMWG), published in 2016, represents the reference document on MRD in MM^2^. However, since its publication, new data have become available, and it is of interest to discuss what other information beyond that included in the IMWG criteria should be captured in ongoing clinical trials (Table 1).

**Table 1:** MRD in multiple myeloma

Validated points	Open issues
MRD negativity is a surrogate for PFS	Optimal threshold for PFS and/or OS prediction by NGS or NGF
MRD negativity is a surrogate for OS	Need for both NGS and NGF
MRD by NGS is standardized	Time interval to define sustained MRD negativity
MRD by NGF (Euroflow) is standardized	Definition of loss of MRD-negative status
MRD by NGS or NGF and PET-CT are complementary	Optimal timing for MRD assessment during and after treatment
MRD useful to compare treatment options	Meaning of MRD negativity in specific subgroups, i.e., high-risk cytogenetics
	Standardization of MRD by PET-CT
	Best tracer for PET-CT
	MRD to alter therapy: duration of maintenance, change treatment, add agents…
	Blood-based MRD assessment
	MRD and detection of clonal evolution
	MRD and MGUS-like profile
	MRD as a valid end-point for drug approval

Minimal residual disease certainly matters in MM. Munshi et al. recently published a meta-analysis on 496 patients in complete response (CR), in whom an MRD-negative status was associated with a significant improvement in both progression-free survival (PFS) and overall survival (OS)^3^. These findings were recently confirmed by the Spanish group in a pooled analysis of three PETHEMA/GEM clinical trials involving 609 patients, showing that MRD-negative status surpassed the prognostic value of CR achievement for PFS and OS^4^. In the paper by the Spanish group and in the majority of the trials included in the meta-analysis by Munshi et al. MRD was mostly assessed by flow cytometry, with a sensivity level of 10^−4^ on average. In the IMWG consensus paper, the definition of MRD negativity requires a minimum sensitivity of 1 in 10^5^ nucleated cells or higher both for flow and sequencing technology. The NGS technology, which is quite well standardized, routinely reaches a sensitivity level of 10^−6^ ^1^. The NGF technology, may easily reach a sensitivity level of 10^−5^, if not 10^−6^, when using the standardized EuroFlow approach^1, 5^.

Therefore, an interesting question is whether a higher level of sensitivity will result in a better predictability, and whether we should try to routinely increase the depth of MRD detection to 10^−6^. In the French IFM 2009 study^6^, which compared RVD versus RVD plus autologous stem cell transplantation (ASCT), MRD was evaluated both by 7-color flow cytometry in all patients and by NGS where possible. Minimal residual disease negativity evaluated by flow was associated with a PFS and OS benefit (sensitivity level of 10^−4^). Of note, among flow-negative cases, the NGS technology was associated with a higher sensitivity (10^−6^) and allowed the segregation of patients into two groups: flow-negative, NGS-negative and flow-negative, NGS-positive, with a significantly worse PFS outcome in the latter population^7^. These results indicate that 10^−6^ might be the ideal cut-off for the definition of MRD negativity. This is even more plausible when the number of patients reaching 10^−6^ in this study was 80 out of 131 evaluable patients^7^. A sensitivity threshold is informative and meaningful when it can be reached by a significant number of patients in a specific therapeutic strategy.

The next question is: NGS or NGF? NGS is now standardized, but the EuroFlow consortium recently described a novel NGF approach using an optimized 2-tube 8-color antibody panel for highly sensitive (close to 10^−6^) and standardized MRD detection that could be implemented in routine diagnostic procedures. In a small number of samples, a comparison of the two techniques showed a good correlation in the percentage of residual abnormal plasma cells detected, with a similar sensitivity^5^. In addition, the EuroFlow technology was recently evaluated in the prospective EMN02 trial, which compared ASCT to bortezomib-based conventional therapy without ASCT and showed a significant impact of flow negative MRD on PFS^8^. Overall, these data indicate that both techniques may be used to evaluate MRD, despite some differences in terms of applicability, availability, cost, sampling, or cell characterization.

What about the role of imaging for the assessment of MRD in 2017? New data on the role of PET-CT have recently been published. In the IFM2009 study^6^, the prognostic impact of PET-CT was convincingly demonstrated^9^. These data were achieved in the context of a prospective study using RVD, which is one of the most effective combinations upfront, and they confirm the prognostic impact of PET-CT already described by the Little-Rock^10^ and Bologna^11^ groups. Another important piece of information provided by this study concerns the complementary role of PET-CT and flow cytometry. A subgroup of patients was evaluated by both PET-CT and by 7-color flow cytometry. Overall, the concordance between the two techniques was low. Progression-free-survival was significantly higher for the group of patients with both a normalized PET-CT and negative MRD by flow versus patients with either PET positivity and/or MRD positivity. When using a Cox model to analyze the impact of a normalized PET-CT, negative MRD and their interaction, the only remaining factor was the interaction, indicating that these two tools may be complementary in predicting patient outcome. Indeed, although we strongly support the use of PET-CT for the evaluation of metabolic response to therapy, it is important to emphasize that both false negative and false positive results may be seen. The Little Rock group recently found that almost 10% of newly diagnosed MM patients had a false negative PET imaging at diagnosis^12^, indicating that new, more sensitive PET-CT tracers, or other imaging modalities, such as whole body diffusion weighted magnetic resonance imaging, should be investigated in the future. Moreover, attempts to standardize FDG PET/CT interpretation criteria are ongoing^13^.

In addition, other important questions remain unsolved. One relates to the concept of sustained MRD negativity. The IWMG consensus paper proposed the confirmation of NGS/NGF and PET negativity a minimum of one year apart^2^. This point is of utmost importance in order to define rules for stopping treatment (during maintenance for example), or to introduce the concept of cure, but, as mentioned by Kumar et al, the definition of sustained MRD negativity was arbitrarily made^2^. However, the number of required monitorings of MRD negativity and the time interval between them should be defined prospectively. This is also true for the new definition of relapse in the IMWG manuscript:^2^ ‘relapse from MRD negativity, that is loss of MRD-negative status with evidence of clonal plasma cells on NGS or NGF, or positive imaging study for recurrence of MM’. What exactly is « loss of MRD-negative status »: a change from 10^−6^ to 10^−5^, 10^−5^ to 10^−4^? Do we need confirmation on two consecutive samples or is one increment sufficient to define relapse? What are the clinical implications of this finding: resumption of interrupted treatment, change of therapy, careful observation in case of absence of biochemical or clinical progression? Indeed, the definition of “loss of MRD-negative status”, which needs clarification, will also impact the new definition of disease-free survival proposed in 2016, which is the duration from the start of MRD negativity to the time of reappearance of MRD^2^. The optimal timing for MRD assessment also remains to be defined.

Overall, MRD assessment will become key in the follow-up of patients with MM. Experts are in agreement that MRD negativity is one of the best prognostic markers, a surrogate for PFS and OS. It is hoped that ongoing (Table 2) and future trials will help to define the optimal use of the technologies to assess MRD, which will potentially determine and tailor our therapeutic strategies.

**Table 2:** Examples of ongoing academic trials evaluating MRD with NGS and/or NFG and/or PET-CT

Trial	**Schedule**
Cassiopeia (Intergroupe Francophone du Myélome/HOVON–NCT02541383)	Randomized trial of VTD versus VTD plus Dara (induction and consolidation)
	All patients receive ASCT
	Second randomization to no maintenance versus maintenance Dara
	MRD measurement (by NGF and NGS) will be done at baseline, post induction, post consolidation and then annually
	PET-CT at baseline and post consolidati
FORTE (GIMEMA–NCT02203643)	Randomized trial of CRd versus CCyd (induction 4 cycles followed by ASCT followed by consolidation 4 cycles) versus CRd 8 cycles without ASCT
	Second randomization to maintenance Lenalidomide versus maintenance Lenalidomide/Carfilzomib
	MRD measurement (by NGF and NGS) will be done at baseline, post induction (4 cycles), pre maintenance and then annually
	PET-CT at baseline, post induction (4 cycles) and pre maintenance
GEM Menos 65 (PETHEMA – NCT 01916252 & NCT 02406144)	Randomized trial of Mel200 versus Bu-Mel as conditioning regimen to ASCT following 6 cycles of induction with VRD
	All patients receive consolidation with 2 cycles of VRD
	Second randomization to Lenalidomide-dexamethasone maintenance versus Lenalidomide-dexamethasone-Ixazomib maintenance
	MRD measurement by NGF will be done at baseline, post induction, post ASCT, post consolidation and then annually
	Based on NGF results following 2 years of maintenance, decision to stop maintenance in case of sustained-MRD negativity, or prolong maintenance 3 additional years in case of NGF positivity

*VTD* bortezomib, thalidomide, dexamethasone, *ASCT* autologous stem cell transplantation, *Dara* daratumumab, *CRd* carfilzomib-lenalidomide-dexamethasone, *CCyd* carfilzomib-cyclophosphamide-dexamethasone, *VRD* bortezomib-lenalidomide-dexamethasone, *Mel 200* melphalan 200mg/m^2^, *Bu-Mel* busulfan-melphalan, *Len-dex* lenalidomide-dexamethasone, *Len-dex-ixa* lenalidomide-dexamethasone-ixazomib, *Neg* negative, *Pos* positive
